# The contribution of microbial biotechnology to sustainable development goals: microbiome therapies

**DOI:** 10.1111/1751-7915.12752

**Published:** 2017-07-11

**Authors:** Paul W. O'Toole, Max Paoli

**Affiliations:** ^1^ School of Microbiology, and APC Microbiome Institute University College Cork Cork Ireland; ^2^ The World Academy of Sciences Strada Costiera 11 34151 Trieste Italy

## Abstract

Complex communities of microbes live on and in plants, humans and other animals. These communities are collectively referred to as the microbiota or microbiome. Plants and animals evolved to co‐exist with these microbes. In mammals, particular kinds of alteration of the microbiome (dysbiosis) are associated with loss of health, most likely due to loss of microbial metabolites, signalling molecules, or regulators of host pathways. Modern life‐style diseases such as Inflammatory Bowel Disease (IBD), Irritable Bowel Syndrome (IBS), type 2 diabetes, obesity and metabolic syndrome have been linked to dysbiosis. These multifactorial diseases involve multiple risk factors and triggers, depletion of certain gut microbiota species being one of them. Live Biotherapeutics operate by restoring microbial products or activities in affected subjects. They are being developed as adjuncts, alternatives or new treatment options for diseases that affect a growing proportion of global citizens.

## Preamble

Complex communities of microbes live on and in plants, humans and other animals. These communities are collectively referred to as the microbiota or microbiome. Plants and animals evolved to co‐exist with these microbes. In mammals, particular kinds of alteration of the microbiome (dysbiosis) are associated with loss of health, most likely due to loss of microbial metabolites, signalling molecules or regulators of host pathways. Modern lifestyle diseases such as inflammatory bowel disease (IBD), irritable bowel syndrome (IBS), type 2 diabetes, obesity and metabolic syndrome have been linked to dysbiosis. These multifactorial diseases involve multiple risk factors and triggers, depletion of certain gut microbiota species being one of them. Live Biotherapeutics operate by restoring microbial products or activities in affected subjects. They are being developed as adjuncts, alternatives or new treatment options for diseases that affect a growing proportion of global citizens.

## SDG(s) addressed

Using microbiota for therapeutic purposes contributes directly to Sustainable Development Goal (SDG) no. 3, ‘Good Health and Well‐being’. In fact, live biotherapeutics products (LBPs) can serve either as the main therapeutic or as an ‘add‐on’ integrating factor; thus, they act by restoring health or helping and assisting to improve general well‐being. Given that a great deal of interconnections and interdependence exist between SDGs, improvements of human health are also associated with reduction in poverty (SDG 1, ‘No Poverty’). In addition, LBPs promote novel approaches to treatment and in the medical sciences in general, leading to innovation in the pharmaceutical industrial sector, which is relevant to SDG 9 (‘Industry, Innovation and Infrastructure’). Finally, the concept of the microbiome puts forward a new way of thinking about the interaction between bacteria and the human body: these insights are overturning previous assumptions, thereby endowing knowledge and endorsing high‐level education (SDG 4, ‘Quality Education’).

## Microbiome therapy solution – live biotherapeutics

A LBP is defined by the relevant US regulatory body, the Food and Drug Administration, as ‘a biological product that: (i) contains live organisms, such as bacteria; (ii) is applicable to the prevention, treatment or cure of a disease or condition of human beings; and (iii) is not a vaccine’ (http://www.fda.gov/downloads/BiologicsBloodVaccines/GuidanceComplianceRegulatoryInformation/Guidances/General/UCM292704.pdf). LBPs are conceptually similar to probiotics (Hill *et al*., 2014), but they differ in having no association with food, either as an isolation source or as a delivery vehicle; they do not have the Generally Recognized as Safe (GRAS) or Qualified Presumption of Safety (QPS) status that many probiotics have; their route to market involves a clinical trial/pharmaceutical regulation pathway like that applied to a new drug. For a review of LBP definitions and regulatory considerations, see a recent review (O'Toole *et al*., 2017).

Live biotherapeutics products, as stated above, are considered to confer clinical benefit primarily by rectifying the consequences of microbiota alterations. For example, patients with IBD have a gut microbiome characterized by depletion or over‐abundance of specific taxa compared to healthy controls(Pascal *et al*., 2017). Based on observations that Clostridia group IV and XIVa species in particular are less abundant in patients with IBD (Manichanh *et al*., 2006), one such organism *Faecalibacterium prausnitzii* is being investigated as a candidate LBP for IBD, following up on promising findings in pre‐clinical colitis models (Sokol *et al*., 2008).

Live biotherapeutics products offer several features that are consonant with SDG principles and practices and that confer advantages over other therapeutic options. LBPs are derived from the microbiome, and many will be administered with a view to restoring ecological deficiencies in the microbiome. They could potentially provide long‐term cures, exemplified by the high rates of long‐term clinical success provided by faecal microbiota transplantation (Petrof and Khoruts, 2014), a much more extreme ecosystem restoration than LBP consumption. In some diseases like IBD and IBS, successful LBP development would eliminate the need for administering therapeutics like corticosteroids and selective serotonin receptor antagonists, respectively, that have broad side effects on host physiology. LBPs may also be useful for restoring a normal microbiome interaction network in diseases characterized by an altered microbiota like IBS (Jeffery *et al*., 2012), or in life stages such as ageing (http://www.nu-age.eu/), or during life events such a stress (http://www.mynewgut.eu/).

## State of the art

The explosion of interest in the microbiome as a determinant of human health has been mirrored by the establishment of dozens of start‐up companies, some allied with multinational pharmaceutical companies, seeking to develop LBPs (https://www.cbinsights.com/blog/microbiome-startups-market-map-company-list/; reviewed in ref.'s (Olle, 2013; O'Toole *et al*., 2017)). Some LBPs are being developed as members of artificial consortia, e.g. bacterial spores that aim to prevent *Clostridium difficile*‐associated diarrhoea (http://www.dilworthip.com/the-emergent-microbiome-a-revolution-for-the-life-sciences-part-i-rd-leaders/), or a mixture of Clostridia intended to inhibit inflammation (http://www.patentdocs.org/2016/11/guest-post-the-emergent-microbiome-a-revolution-for-the-life-sciences-part-viii-the-microbiome-and-i.html). Administering consortia is technically challenging but is concordant with SDG principles because it attempts to restore or partly restore the original ecosystem balance. Single LBPs being considered, in development or under evaluation, include *Blautia hydrogenotrophica* for IBS (http://www.researchandmarkets.com/reports/3961131/irritable-bowel-syndrome-pipeline-review-h2#), *Eubacterium hallii* for metabolic disease (Udayappan *et al*., 2016), *Lactobacillus reuteri* for type 2 diabetes (Mobini *et al*., 2017) and *Bacteroides fragilis* for autism (Abdollahi‐Roodsaz *et al*., 2016). As stated above, many LBPs under development are intended to restore the microbial ecological network that is typical for a body site in a healthy individual. For example, C3J therapeutics is developing therapeutics that prevent colonization of the oral cavity by oral pathogens while favouring the colonization by microbes associated with oral health (https://www.cbinsights.com/blog/microbiome-startups-market-map-company-list/). In a different approach, second genome is using analysis of microbiota alterations and host cellular/transcriptional responses in patients with IBD to identify targets and small molecules to treat IBD (Abdollahi‐Roodsaz *et al*., 2016).

Notwithstanding the excitement and general optimism of microbiome research, it is important to note in a state‐of‐the‐art evaluation that microbiome research is still in a very early stage and that therapeutics derived directly from microbiome research are not yet in the marketplace for reasons discussed below.

## Obstacles to solutions

The main obstacle to LBP development, which is actually more of a scientific and project management challenge, is to produce good quality phase 1 and phase II trials to demonstrate safety, tolerance and clinical efficacy. In contrast to testing small molecules through well‐established production practices and trial designs, taking LBPs through the same process is more difficult because of the technological features of the organisms (reviewed in O'Toole *et al*., 2017). In brief, many of the organisms of interest are strict anaerobes which requires extensive modification of production processes including exclusion of oxygen from freeze‐drying, formulation and (if required) encapsulation, all of which must be performed according to good manufacturing practice so that the product is suitable for human consumption. Placebo product for controlled trials must be available and must be indistinguishable to the study participants. The study population must be representative of the disease being targeted, and it may be desirable to at least profile participants’ microbiota at baseline, if not stratify patients by microbiota, to inform evaluation of clinical responses.

## Contribution of microbiome therapies

Microbiome therapies and LBPs have opened opportunities and perspectives that are clearly aligned with the directions of sustainable development. Their impact on SDG 3 – ‘Good Health and Well‐being’ – is significant as articulated in the previous sections. In addition, these new tools are providing a different approach for professionals such as researchers, medical doctors, clinicians and pharmaceutical experts alike. Interestingly, this angle has the potential to change the mindset of workers in the field as well as anyone involved in high‐level professional development and education. The teaching of microbiome therapies and their underlying concepts means that the human body will be considered more carefully not as a single organism but as a host of a whole community with equilibrium and balance. This is in line with the philosophy of sustainability rooted throughout the 169 targets of the SDGs in the United Nations’ 2030 Agenda. The contribution of microbiome therapy research and related advances such as LBPs are likely to have a ripple effect on a number of topical fora relevant to the SDGs like, for example the ‘One Health Initiative’ (http://www.onehealthinitiative.com/), ecosystem services and sustainable medicine.

## Competing‐complementing non‐microbial biotech strategies

Amongst the plethora of non‐microbial biotechnology tools, molecular biotechnology and chemical techniques dominate the scene. However, microbiome therapy research findings have flagged the importance and advantages of looking beyond molecules and even beyond cells and organisms: the idea is to strive towards an all‐encompassing view of the microbiome. Microbiome therapies and LBPs may provide strategies that are more amenable to sustainable development in that they are mindful of the natural state of the body and work on the maintenance or re‐establishing of the equilibrium of the microbiome. The rational approach used for microbial strategies and deriving from microbiome therapies in particular mirrors the vision portrayed in the SDGs: ‘Technological progress in harmony with nature’ (UN 2030 Agenda; http://anhinternational.org/2015/09/30/united-nations-sustainable-development-goals-better/).

## Concluding remarks

The human microbiome and microbiome therapies in particular have changed the way in which we think about individual plants and animals. Microbiome therapies bring about a novel approach to treatment and to the way in which we have conceived the human body. The new philosophy of our body as a collection of populations hosted by each one of us in a functional equilibrium establishes a holistic vision with repercussions on health as well as education. A strong principle distilled out of the microbiome research findings over the last decade is that of balance.

All these concepts are also key pillars of the ideology of sustainability put forward by the United Nations’ new 2030 Agenda. It is, in fact, not possible to work towards sustainable development without embracing the need for equilibrium and balance, which are essential to the environment and Earth's resources, and also extremely relevant, as shown by microbiome research, to the functioning of the body.

The holistic vision of single human beings being the cradle of complex communities of microorganisms is in line with the rationale of sustainability: we ought to consider humans in the same way as the planet, i.e. as hosts of delicate balance and intricate interdependence. In turn, we as human individuals depend on the proper maintenance of the ‘microbiome interactome’ for good health and general well‐being.

As part of the continuing efforts towards education for sustainable development (ESD) by the UN and UNESCO, training of scientists and education of the wider public must include key concepts such as the ones flagged here if we are to catalyse the change of mind required for a sustainable future.

## Conflict of interest

None declared.
